# Study protocol: the Fueling Learning through Exercise (FLEX) study – a randomized controlled trial of the impact of school-based physical activity programs on children’s physical activity, cognitive function, and academic achievement

**DOI:** 10.1186/s12889-016-3719-0

**Published:** 2016-10-13

**Authors:** Catherine M. Wright, Paula J. Duquesnay, Stephanie Anzman-Frasca, Virginia R. Chomitz, Kenneth Chui, Christina D. Economos, Elizabeth G. Langevin, Miriam E. Nelson, Jennifer M. Sacheck

**Affiliations:** 1 Gerald J. and Dorothy R. Friedman School of Nutrition Science and Policy Tufts University, 150 Harrison Avenue, Boston, MA 02111 USA; 2 Department of Pediatrics, Jacobs School of Medicine and Biomedical Sciences, University at Buffalo, G56 Farber Hall, South Campus, Buffalo, NY 14214 USA; 3 Department of Public Health and Community Medicine, Tufts University School of Medicine, 136 Harrison Avenue, Boston, MA 02111 USA; 4 ChildObesity180, Tufts University, Boston, MA USA; 5 Sustainability Institute at the University of New Hampshire, 107 Nesmith Hall, 131 Main Street, Durham, NH 03824 USA

**Keywords:** School children, School-based physical activity intervention, Executive function, Childhood obesity, Health disparities

## Abstract

**Background:**

Physical activity (PA) is critical to preventing childhood obesity and contributes to children’s overall physical and cognitive health, yet fewer than half of all children achieve the recommended 60 min per day of moderate-to-vigorous physical activity (MVPA). Schools are an ideal setting to meeting PA guidelines, but competing demands and limited resources have impacted PA opportunities. The Fueling Learning through Exercise (FLEX) Study is a randomized controlled trial that will evaluate the impact of two innovative school-based PA programs on children’s MVPA, cognitive function, and academic outcomes.

**Methods:**

Twenty-four public elementary schools from low-income, ethnically diverse communities around Massachusetts were recruited and randomized to receive either *100 Mile Club®* (walking/running program) or *Just Move™* (classroom-based PA program) intervention, or control. Schoolchildren (grades 3–4, approximately 50 per school) were recruited to participate in evaluation. Primary outcome measures include PA via 7-day accelerometry (Actigraph GT3X+ and wGT3X-BT), cognitive assessments, and academic achievement via state standardized test scores. Additional measures include height and weight, surveys assessing psycho-social factors related to PA, and dietary intake. School-level surveys assess PA infrastructure and resources and intervention implementation. Data are collected at baseline, mid-point (5–6 months post-baseline), and post-intervention (approximately 1.5 years post-baseline). Demographic data were collected by parents/caregivers at baseline. Mixed-effect models will test the short- and long-term effects of both programs on minutes spent in MVPA, as well as secondary outcomes including cognitive and academic outcomes.

**Discussion:**

The FLEX study will evaluate strategies for increasing children’s MVPA through two innovative, low-cost, school-based PA programs as well as their impact on children’s cognitive functioning and academic success. Demonstration of a relationship between school-based MVPA with neutral or improved, rather than diminished, academic outcomes in a naturalistic environment has the potential to positively influence investment in school PA programs and initiatives.

**Trial registration:**

ClinicalTrials.gov Identifier: NCT02810834. Registered May 11, 2015. (Retrospectively registered)

## Background

Physical activity (PA) plays a key role in childhood obesity prevention, in addition to conferring a number of other health benefits [1–4]. Yet fewer than half of all children in the U.S. meet the recommended 60 min of daily moderate-to-vigorous physical activity (MVPA) [5]. Schools are an ideal setting to achieve maximum impact with respect to improving PA levels, given the significant amount of time children spend in school over the course of their childhood [6]. Yet competing demands on teachers’ time, a crowded school curriculum, increased focus on standardized tests, and constrained school budgets have limited PA programming in schools [7, 8]. However, a growing body of evidence demonstrates that school-time PA is positively associated with academic achievement [9–13].

Novel strategies are needed to increase PA opportunities for children during school time. Recently, experts have called for a “whole school” approach to increasing children’s PA levels [14] in which recess, in-class PA breaks, before- and after-school programs, and integration of PA with academic curricula combine to create healthy school environments. Taken together, this comprehensive approach can increase time children spend engaging in MVPA to meet the recommendation of 60 min per day, 30 of which should be accrued during school hours.

Emerging evidence suggests that this “whole school” approach may be even more critical for underserved children. Compared to their higher-socio-economic status (SES) peers, children from low-income communities get a greater proportion of their total daily PA in school [15]. However, environmental barriers, such as limited PA-supporting policies, activities, and infrastructure, have been observed in lower-SES schools [16–18]. Under-resourced schools may face significant constraints to implementing school-based PA programs to supplement physical education (PE), thereby exacerbating disparities in PA, obesity, and academic achievement. Even short bouts of activity may predict determinants of future engagement in PA, suggesting that small increases in school-time MVPA could lead to additional increases in total daily MVPA and concomitant improvements in physical health and academic outcomes in underserved children [14, 19, 20].

Longitudinal evidence of the effects of school-based PA programs on physiological, behavioral, and academic outcomes is limited for racially diverse schoolchildren [14], and few studies have evaluated the impact of changes in MVPA on standardized test scores in this population in the context of a randomized school-based PA intervention study. The Fueling Learning through Exercise (FLEX) Study is a randomized controlled trial that seeks to evaluate the impact of two innovative school-based PA programs not only on MVPA, but also cognitive and academic outcomes over time among 3rd-5th grade children in underserved schools in northeastern United States. The primary aim of this paper is to describe the design and study protocol of the FLEX Study.

## Methods/Design

### Aims

The primary aim of the FLEX Study is to evaluate the impact of two school-based PA programs, *100 Mile Club®* and *Just Move™*, on children’s school-time MVPA and total daily MVPA, compared to a control group. In addition, the RCT will evaluate the effects of these innovative, school-based PA programs on children’s cognitive performance and academic achievement. The study will also evaluate the reach of the programs by examining factors that influence participation among different demographic, weight status, and fitness groups.

## Study design - overview

**Fig. 1:**
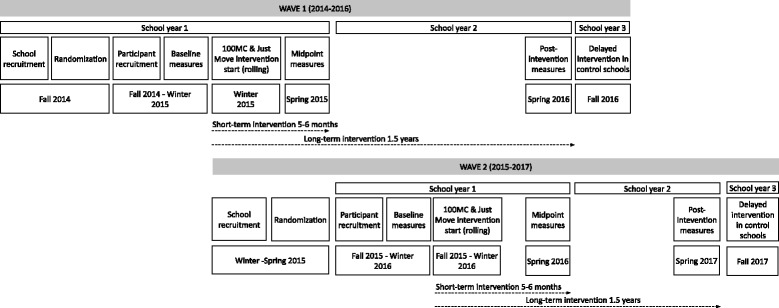
Timeline for the FLEX study

The FLEX Study is a cluster randomized controlled trial. Participating schools were clustered by location within the state and school district. Schools within each district were randomly assigned to receive a school-based PA intervention (*100 Mile Club* or *Just Move*) or assigned to be a control school. Third and fourth grade students were enrolled and are being followed for two school years (Fig. 1). Data are collected at three time points: baseline (fall, school year 1), short-term (spring, school year 1; 5–6 months post-baseline) and long-term post-intervention (spring, school year 2; ≈1.5 years post-baseline). The study occurred in two waves, the first beginning during the 2014–2015 school year (*n* = 6 schools) and the second wave during the 2015–2016 (*n* = 18 schools) school-year. Due to unforeseen weather circumstances and school cancellations in Winter 2015, additional student participant recruitment beyond the initial six schools was put on hold until the following school year. The investigative team determined that these schools would be designated as Wave 1. Approval for the study was obtained from the Tufts University IRB (Medford, MA) and additionally from individual school districts where required.

## Setting & participants

### Schools

Identification of eligible schools began at the district level. The FLEX study is designed to examine health disparities among children associated with income and race/ethnicity. Therefore we targeted lower income and racially/ethnically diverse public school districts in Massachusetts. Initially, school districts were approached for participation if they had greater than 40 % of students qualifying for free or reduced price lunch and/or had greater than 40 % non-Caucasian students. In addition, districts were only approached if they had at least four elementary schools serving 3rd-5th graders.

Overall, twenty school districts were approached. Twelve of these districts declined to participate, citing changes in administration, ongoing participation in similar programs, and/or competing priorities. From the remaining eight districts, 24 schools agreed to participate and have at least one school enrolled. Six schools were included in Wave 1 and 18 schools in Wave 2. All districts serve a low-income and diverse population and are located in urban, suburban, and peri-urban areas. One exception to the initial inclusion criteria was made, such that one school from a district with 34 % low-income students was included.

### Students

All third and fourth grade students at participating schools were eligible to enroll. Third and fourth grade students were recruited in year one and followed in year two into fourth and fifth grade, respectively.

## Recruitment

### School recruitment & randomization

**Fig. 2:**
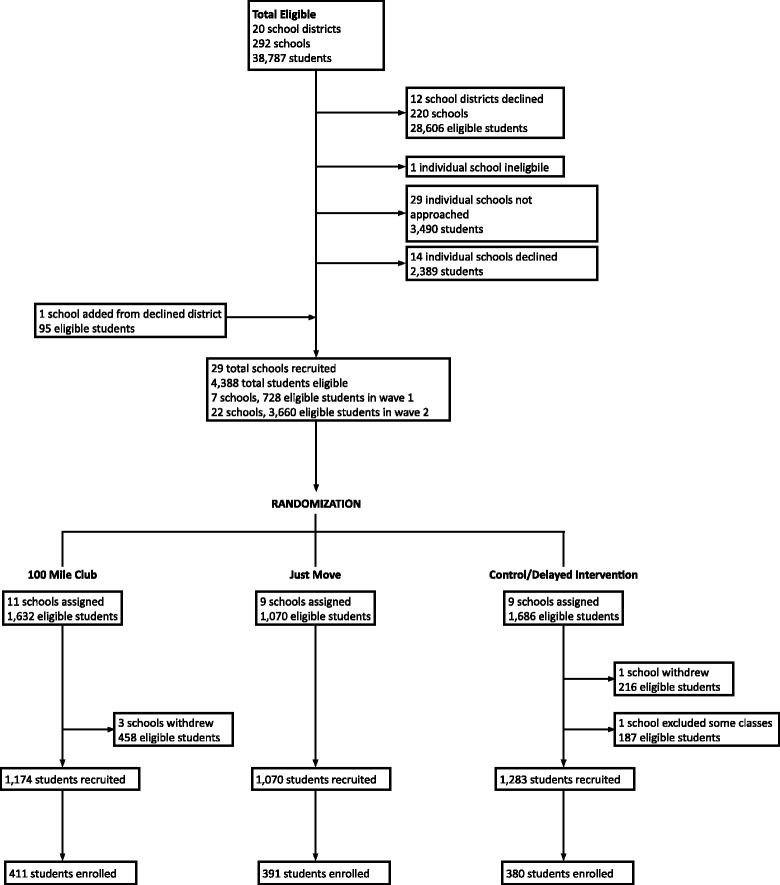
District, school, and participant recruitment diagram for the FLEX study

Schools were initially recruited by email and phone, followed with in-person meetings. Initial contact was made at the district level to superintendents and district wellness/health/PE coordinators, with secondary outreach at the individual school level (principals/assistant principals). Where districts have research boards/IRBs, contact was simultaneously made to those entities, and the study protocol and recruitment materials were submitted for approval. Once enrolled, schools were randomized to receive one of two school-based PA programs – *100 Mile Club* or *Just Move* – or to the control group. Schools were block randomized in groups of three, stratified by district, to ensure comparable numbers of schools in each arm. Once a school agreed to participate, the study statistician informed recruiting staff of the school’s group assignment. Throughout the school recruitment process, the statistician and recruitment staff worked independently. Figure 2 outlines the flow of district, school, and participant recruitment.

## Interventions

The *100 Mile Club* is a school-based program that encourages children to walk, run, or wheel 100 miles over the course of the school year (approximately 3 miles per week). The program can be implemented before, during, and/or after school depending on the school schedule and is led by one or two champions (e.g., PE teachers), identified by school administration, who log student miles. Champions are encouraged to tally participants’ miles each week and display participant and school progress in a prominent location. The goal is to encourage participants and provide positive feedback and reinforcement. Champions are trained by study staff and are provided materials, resources, and ongoing support for implementation throughout the duration of the intervention.

*Just Move* is a program of structured classroom-based, PA breaks that integrates both high- and low-intensity movements (ex. jumping jacks, squats, stretches, yoga) with academic material to provide children with opportunities for engaging in PA while learning. Breaks are designed to be short (5–15 min), and teachers are encouraged to incorporate at least one break per day. *Just Move* activities are presented on cards with a picture of the movement and suggestions for connecting the moves with academic subjects like math, language arts, and science. Study staff train teachers to implement the activity breaks and provide a set of cards along with strategies and ideas for making the breaks fun and engaging for their students and low-burden for the teachers themselves. Study staff provide ongoing support to classroom teachers throughout the intervention to help ensure that teachers continue to implement *Just Move* breaks in their classrooms.

Both the *100 Mile Club* and *Just Move* program originated in schools and were identified by the Active Schools Acceleration Project (ASAP), a nationwide initiative dedicated to increasing the PA of U.S. schoolchildren [21, 22]. The *100 Mile Club* and *Just Move* were selected through a nationwide contest for school-based PA innovation in 2012, assessed for scalability and implementation potential, and currently take place in schools around the U.S. Both programs are low-cost, require minimal resources to implement, and are flexible and adaptable to a range of school environments. Teacher-developed and champion-led school-based PA programs may have unique advantages with respect to feasibility and sustainability, as opposed to programs developed by researchers outside of the school environment [23].

Control schools will receive a delayed intervention of either the *100 Mile Club* or *Just Move* after completion of the study (Wave 1 in Fall 2016; Wave 2 in Fall 2017).

### Student recruitment

Study staff conducted presentations in schools to explain the study and enrollment procedures, and to distribute recruitment materials to all 3rd and 4th grade students. Presentations were given assembly-style or in individual classrooms. Permission packets were sent home with each student and included: 1) a flyer with information about data collection procedures; 2) a plain language consent form for the parent/guardian; 3) a child assent form; and 4) a demographic form for the parent/guardian to complete. Permission packets were available in English, Spanish, Portuguese, Haitian Creole, Arabic, Vietnamese, and Mandarin, the primary languages spoken in targeted communities. Students were asked to return completed packets approximately 1 week after they were distributed. Enrollment was closed at the start of baseline data collection.

## Data collection methods

### Overview

Detailed information about study measures and time points is presented in Table 1.

**Table 1:** Data collection plan for the FLEX Study

	Baseline (School Year 1)	Midpoint (School Year 1)	Post-Intervention (School Year 2)	Once per school year
Participant level measures				
Primary outcome measures				
Physical activity (accelerometry)	√	√	√	
Cognitive function (Digit Span and Stroop Color-Word tests)	√	√	√	
Standardized test scores	√^a^	√	√	
Attendance	√^a^	√	√	
Anthropometric measures				
Height	√	√	√	
Weight	√	√	√	
Additional physical activity measures				
Social support for PA	√	√	√	
What Kind of Kid Are You? (self-perception of behavior, athletic competence and global self-worth)	√	√	√	
Cardiorespiratory fitness (PACER)				√
Dietary measures				
Weekday breakfast consumption	√	√	√	
7-day dietary recall	√	√	√	
Demographic measures				
Date of birth	√			
Grade	√			
Sex	√			
Race/Ethnicity	√			
Free/reduced price lunch eligibility	√			
Behavioral/developmental difficulties	√			
Individualized education program status	√			
Maternal education level	√			
Paternal education level	√			
School-level measures				
School physical activity environment scan				√
School food environment scan				√
Program evaluation measures (100 Mile Club and Just Move schools)				
Participant-level program participation and attitudes		√	√	
Champion/teacher program questionnaire	√	√	√	
Direct observation				√

^a^Collected in the spring of the prior year

Briefly, child-level data collection takes place at each participating school during regular school hours at each of the three time points (baseline, midpoint, and post-intervention). Exceptions are demographic data, which are collected once at baseline, and fitness measurements, which are conducted at a separate time once per school year. Child-level standardized test scores for Math and English Language Arts (ELA) are collected from the Massachusetts Department of Elementary and Secondary Education (DESE) after testing is completed each spring. School-level data is collected once per year (physical activity environment and food environment surveys, along with attendance data). Trained research assistants (RAs) and study staff administer all instruments and assessments according to the written study protocol. At baseline, there were 174 participants enrolled from Wave 1 schools and 1008 from Wave 2 schools. Baseline measurements were conducted in the fall/early winter of Year 1 (Wave 1 – February-March, 2015; Wave 2 – September 2015-February 2016). Short-term measurements were conducted at the end of the first school year (Wave 1 – May-June 2015; Wave 2 – April-June 2016) and post-intervention measurements will be conducted approximately 18 months after baseline at the end of the second school year (Wave 1 – March-April 2016; Wave 2 – April-June 2017). At time of enrollment, participants are assigned a unique 5-digit code which is used in place of name or other identifiers to link participant to all their study data. The study project manager maintains an electronic, password-protected list linking participant with their unique ID.

### Demographics

Demographic information was collected at baseline by paper-and-pencil questionnaire included with the recruitment packet and returned with informed consent documents. The 10-item questionnaire included questions on child’s date of birth, grade and age at time of enrollment, sex, race/ethnicity, maternal and paternal education levels, and free- or reduced-price lunch status. Parents/guardians were also asked to report whether or not their child has behavioral difficulties, such as learning, understanding or paying attention, or communicating [24] and whether their child was on an individualized education program (IEP). English language learner status will be obtained at the child-level from the Massachusetts DESE.

### Anthropometrics

Height and weight are measured in light clothing with shoes removed using a portable stadiometer (Model 213, Seca Weighing and Measuring Systems, Hanover, MD) and portable digital scale (Model 803, Seca Weighing and Measuring Systems, Hanover, MD). Height and weight are measured in triplicate to the nearest 1/8 in. and 0.1 kg, respectively. Body mass index (BMI) will be calculated (kg/m^2^) and converted into z-score using the Centers for Disease Control and Prevention (CDC) age- and sex-specific growth charts [25]. BMI percentiles are classified accordingly as: <5th percentile as underweight, 5th-85th percentile as normal weight, 85th-95th percentile as overweight, and ≥95th percentile as obese.

## Primary outcomes

### Physical activity

Waist-worn tri-axial accelerometers are used to objectively measure participants’ physical activity (Actigraph accelerometers, models GT3X+ and wGT3X-BT, ActiGraph, LLC, Pensacola, FL) and have been validated and calibrated for use among children [26]. Study staff instruct each participant how to wear the accelerometer. Participants are asked to wear accelerometers during all waking hours, except when bathing or swimming, for 7 consecutive days. Accelerometers are initialized to store activity counts beginning at 00:00:01 on the first day the child will wear the device; data will be processed using a 15-s epoch. To support wear-time compliance, participants are also given a paper calendar-style tracking log on which they are instructed to write down the time they put the accelerometer on each morning and the time they remove it before bed each night. The log also asks participants to self-report any days they were ill during the wear week, as well as any sports or physical activities they played during the wear week and their favorite sports/physical activities. In addition, weather data are collected from the National Oceanic and Atmospheric Administration (NOAA) [27], and the high temperature (continuous) and precipitation (binary: yes/no) are recorded for each day accelerometers are worn. Data are collected from the weather station nearest to the participating school.

Accelerometer data will be categorized into minutes of sedentary, light, moderate and vigorous activity using thresholds developed specifically for children [28]. In-school and out-of-school time MVPA will be extracted. In-school hours will be calculated for each participant based on specific start and end times of the school day for each day the accelerometer was worn. Weekday out-of-school time will be calculated as the sum of before school time and after school time, accounting for school hours and average awake time.

### Cognitive performance

Two cognitive assessments are administered one-on-one in a quiet area. A digit span test, with forward and backward subtests, is used to measure short-term memory, attention and concentration [29]. Digit span tests have been positively linked to PA [29, 30]. In the test, the administrator orally presents number lists of increasing length to the participant, and the participant is asked to repeat these digit spans back in the same order either forward or backward (for forward and backward subtests, respectively.)

A Stroop Color-Word test [31] is administered to assess inhibitory control and selective attention, aspects of cognitive function that have been positively linked with PA and fitness in previous studies [30, 32]. The test includes both a congruent task and an incongruent task, each lasting 45 s. Participants are first given the congruent task in which they are presented with a set of 100 color words printed in the *same* color ink as each word (ex. the word “*red*” is printed in red ink). They are asked to read these words aloud and complete as many as possible in 45 s. After completing this task, they are given the incongruent task, in which they are provided another set of 100 color-words printed in a *different* color ink (ex. the word “*red*” is printed in green ink). Participants are asked to identify and say aloud the color of the ink rather than the word, completing as many as possible in 45 s. The test is scored by calculating a ratio of items completed correctly to total number of items completed in 45 s.

### Academic outcomes

Individual child-level standardized test scores will be collected from the Massachusetts DESE and used as an indicator of academic achievement. PA in children has been shown to be positively associated with scores on both Math and ELA [33, 34]. The Massachusetts Comprehensive Assessment System (MCAS) test has been used in Massachusetts annually for all students in grades 3–10. During the 2014–2015 academic year, the DESE began the process of transitioning to a different standardized test, the Partnership for Assessment of Readiness for College or Careers (PARCC). The MCAS/PARCC tests are administered annually in the spring and coincide with both mid- and post-intervention measurements. Baseline standardized test scores (year prior to enrollment) will be obtained retrospectively and for each year of participation for all participants. For both tests, raw scores are converted to scaled scores, which are linked to four levels that describe the performance levels of individual students, schools, and districts: Advanced, Proficient, Needs Improvement, and Warning. Additionally, grade-level standardized test scores will be collected across grades 3–5, and will be aggregated to analyze school-level impact of *100 Mile Club* and *Just Move*.

School attendance will be collected and examined as an exploratory outcome at the individual and school level. Attendance will be assessed as the number of days present per academic school year and converted to a percentage.

## Potential individual-level covariates

### Physical activity social support and self-efficacy

A PA social support questionnaire is administered to participants in small groups, with individuals completing their own questionnaire. The 10-item questionnaire, developed by Trost and colleagues [35], is modified from the Social Influences Scale developed and validated by Saunders et al. [36]. The modified questionnaire assesses the participant’s self-reported social support for engaging in physical activity by family, friends, classmates and teachers. Questions are answered on a three-point scale (“yes,” “no,” or “don’t know”). Example scale items include “*My mother/father/caretaker thinks I should be physically active*,” “*The students in my class think I should be physically active*,” and “*My teacher has encouraged me to be physically active in the last two weeks.*”

Participants also complete the “What Kind of Kid Are You?” 18-item questionnaire which is designed to assess how children evaluate themselves in various domains including athletic competence, behavioral conduct, and global self-worth. Questions are taken from the Harter Self-Perception Profile for Children [37]. Participants are asked which of two opposing statements best describes them and then whether it is “sort of true” or “really true” for them. This structured alternative format is designed to minimize socially desirable responses and ensure internal consistency and reliability [38].

### Dietary intake

Dietary patterns are collected by child self-report using two instruments, both administered in small groups. One questionnaire focuses specifically on weekday breakfast consumption, and the other is a 7-day recall focused on diet quality. The 6-item breakfast questionnaire developed for this study asks about typical weekday breakfast consumption, including whether or not the participant usually eats breakfast on school days, source of breakfast (school, home, restaurant), and foods and beverages typically consumed for breakfast. The same questions are asked about “today” (the day of data collection). Breakfast consumption has been linked with stronger cognitive functioning in children [39] and data on breakfast consumption will be explored as a potential covariate or moderator of intervention effects on cognition.

Diet quality is assessed through a 39-question 7-day recall. Research assistants explain to participants how to complete the questionnaire. The FLEX Dietary Questionnaire is adapted from several published, validated instruments [40–42]. The recall is divided into 5 categories: beverages, fruits, vegetables, salty snacks, and sweet snacks. The structure is similar to the Block Food Frequency Questionnaire for Ages 8–17 [43] in which respondents indicate how many days in the last week they consumed an item (none, 1 day, 2 days, 3–4 days, 5–6 days, or everyday) and how much they had in one day (a little, some, or a lot). The Block Food Frequency Questionnaire for Ages 8–17 was used during Wave 1, but field administration showed the foods included to be less relevant to the consumption patterns of the study participants and the format (scantron form) to be challenging for the 8–10 year old participants to complete. Foods and beverages included in the FLEX Dietary Questionnaire, administered in Wave 2, reflect the foods and beverages frequently consumed by children in this age group as well as foods and beverages to limit (sugar-sweetened beverages, salty snacks, desserts) and those to promote (fruits, vegetables, water) [44]. The portion size for each item is matched to a standard serving size to represent “a little” (1/2 standard serving), “some” (1 standard serving) and “a lot” (1.5 times standard serving). A final question asks how many days the respondent has eaten out (including restaurants, fast-food, or take-out) in the last 7 days. Each questionnaire receives an overall score for diet quality and will be used as a covariate in analyses.

### Fitness assessment

Cardiorespiratory fitness (CRF) is measured as a covariate using the FITNESSGRAM^®^’s Progressive Aerobic Cardiorespiratory Endurance Run (PACER) test, which is a 20-meter maximal-effort shuttle-run. The 20-meter shuttle-run has been validated with young populations [45] and shown to be highly correlated with VO_2_max in children [45, 46]. The test is administered once per school year (for 2 school/study years) in the study schools by trained research staff who follow a standard protocol based on the Cooper Institute’s published guidelines [47]. Aerobic capacity as measured by the PACER will be analyzed using standards described by FITNESSGRAM [47].

## School level data

The study champion/liaison at each school (ex. principal or other administrator, PE teacher, health coordinator) is asked to complete a short, 14-item online survey, which assesses the PA environment (PAE, including practices and policies). Questions from the PAE were adapted from the School Physical Activity Policy Assessment (S-PAPA) [16, 48]. The survey is divided into sections to assess PA-supporting policies and practices in four areas relevant to the school environment: PE, recess, classroom-based PA, and before- and after-school PA opportunities. Scores on the PAE scan will be tabulated based on policies and practices identified as being related to children’s MVPA during school [16, 49]. Total point scores will also be either median-split into high- and low-PAE or stratified by percentile (low = 10th percentile, medium = 50th percentile, high = 90th percentile) for analysis.

The champion/liaison at each school is also asked complete an online, 14-question survey about the school food environment, policies, and practices. Questions are divided into categories including breakfast, snacks, competitive foods, and other nutrition or healthy eating programming that the school is participating in and were drawn from categories included in school wellness assessment tools [50]. Both the school PAE scan and the school food environment survey are completed once per school year.

Other school-level data collected will include PE schedules and lunch and recess times. These data will be linked to individual participants and potentially included in analyses to explore impact on cognitive outcomes (i.e. whether timing of lunch/recess/PE relative to participants’ completion of cognitive tests relates to performance).

## Process evaluation

To better understand implementation of the intervention programs (*100 Mile Club* and *Just Move*), several process measures are being collected in the intervention schools. Children are asked to complete a short questionnaire at both midpoint and post-intervention to assess program participation, acceptance, and sustainability.

School champions, in both *100 Mile Club* and *Just Move* schools, and classroom teachers implementing the *Just Move* program, are asked to complete brief surveys at baseline, midpoint, and post-intervention. Questions are designed to gather information on frequency and dose of the programming. In addition, questions assess factors related to implementation including facilities and resources used; leadership and modeling of PA behaviors; strategies for engagement; and perceptions of the program by school administration, other staff, parents, and children.

Direct observations of the programming are conducted in each of the intervention schools, once per school year. Using a standard rubric for the observation, study staff will document numbers of children participating, engagement of participants, and resources being used. Communication with champions and classroom teachers about program implementation will be documented systematically.

## Sample size calculation and data analyses

A priori sample size calculations for the FLEX study were based on several recent school-based physical activity interventions. Power calculations for our analysis with respect to school-time MVPA were based on the findings of Verstraete et al. [51]. In this study, the intervention group showed a greater percentage of recess time spent in MVPA, compared to controls (mean ± SD: 53.4 ± 25.6 vs. 43.5 ± 27.6). While we expect a larger difference from our more intensive intervention, we used this conservative estimate to calculate our sample size, resulting in an estimated sample size of 115 participants per arm (total *n* = 345) with power of 80 %. For total daily MVPA, we referred to the findings from a RCT to increase PA through curriculum modification by Donnelly et. al [52]. In this study, the intervention group received 90-min/week of physically active classroom lessons (≈10 min/lesson) which resulted in an increase of 26 min of weekly MVPA in the intervention group compared to controls (within group SDs = 40). We estimated that a sample size of 39 per arm (117 total) is needed to detect differences of this magnitude. Thus, the sample size of 345 will be sufficient. Because the number of clusters is fixed at 21 schools, 7 per arm, we used a protocol suggested by Hemming et al. [53] to adjust for the clustering. Assuming an ICC of 0.03 and attrition rate of 25 % per year, we arrived at a final sample size of 903 students, 43 per school.

During the 2014–2015 study roll out, Boston experienced a severe snow season which impacted the school recruitment process. After consulting with the funding agency we subsequently extended school recruitment and intervention implementation into the next year, creating the two-wave structure described previously.

For statistical analyses, descriptive statistics for variables of interest will be compiled and tabulated. To test the effect of school-based PA programs on minutes spent in MVPA, we will compare *100 Mile Club* vs. control and *Just Move* vs. control as separate tests using mixed effects models. Outcomes of interest are changes in minutes of MVPA between midpoint vs. baseline and post-intervention vs. baseline. The key regression coefficient of interest is the intervention by time interaction term, which will indicate if the intervention is associated with a significant higher/lower change in MVPA across time compared to the control. The unique school identification number will be modeled as random intercepts to adjust for clustering. We will examine both unadjusted and adjusted (including covariates such as sex, race/ethnicity, weight status) regression coefficients of the respective program and conclude if the difference in MVPA minutes is statistically significant. A similar approach will be used to examine the impact of PA programming on academic/cognitive outcomes. All analyses will be performed using SAS 9.3 (Cary, NC, USA) and results with p-values less than 0.05 will be considered statistically significant.

## Discussion

The Fueling Learning through Exercise (FLEX) Study will contribute to the evidence base on strategies for increasing children’s engagement in PA at school by using objective measures of physical activity to evaluate the impact of two innovative, low-cost, school-based PA programs on children’s PA as well as on their cognitive functioning and academic success. Demonstration of a relationship between school-based PA with neutral or improved, rather than diminished, academic outcomes has the potential to positively influence school administrators’ investment in PA programs and initiatives.

The participants in the FLEX Study are from racially- and ethnically-diverse, primarily low-income communities. Rising inequalities in the prevalence of childhood obesity [54] and lack of demonstrated success to increase PA in high-risk children further underscore the need to identify PA interventions with equitable reach [55]. PA programs should reach all children as opposed to making the “fit kids fitter” or failing to impact MVPA in those who are overweight or obese. A recent evaluation of MVPA among inner-city elementary schoolchildren based on accelerometry found substantial disparities, such that MVPA was lower among females, Hispanics, and overweight and obese children [35]. Accelerometry studies based on nationally-representative samples such as NHANES indicate an even more complex relationship between PA and gender, weight status, and race/ethnicity [56]. Belcher et al. found that while obese youth were generally less active, this did not hold true across all racial/ethnic groups. These findings, and others, suggest that social and environmental supports may be critical ecologic contributors to reducing disparities observed in MVPA among underserved youth [57, 58].

However, disparities in PA-supporting policies and practices, such as lower-SES schools being less likely to have PE teachers and fewer supporting PE practices compared to higher-SES schools, may further undermine the ability of underserved youth to achieve recommended PA levels [17]. These findings indicate an even greater need for structured recess as well as before- and/or after-school and classroom-based PA opportunities to supplement PE time in these environments, and further suggest that school-based PA programs have the potential to attenuate disparities. Understanding the effects of such programs over time and correlates of participation in them among diverse schoolchildren at high risk of obesity is essential to the design and expansion of effective interventions with the potential to achieve a broad reach and attenuation of health disparities, and support children in developing healthy lifestyle habits.

## Abbreviations

BMI: Body mass index
CRF: Cardiorespiratory fitness
DESE: Department of Elementary and Secondary Education
ELA: English Language Arts
FLEX: Fueling Learning through Exercise
ICC: Interclass correlation coefficient
IRB: Institutional Review Board
LPA: Light physical activity
MCAS: Massachusetts Comprehensive Assessment System
MVPA: Moderate-to-vigorous physical activity
PA: Physical activity
PACER: Progressive Aerobic Cardiorespiratory Endurance Run
PAE: Physical activity environment
PARCC: Partnership for Assessment of Readiness for College and Careers
PE: Physical education
RA: Research assistant
RCT: Randomized controlled trial
SES: Socio-economic status

## References

[1] Andersen LB, Riddoch C, Kriemler S, Hills A (2011). Physical activity and cardiovascular risk factors in children. Br J Sport Med.

[2] Janssen I, LeBlanc AG (2010). Systematic review of the health benefits of physical activity and fitness in school-aged children and youth. Int J Behav Nutr Phys Act.

[3] Kriemler S, Zahner L, Schindler C, Meyer U, Hartmann T, Hebestreit H, Brunner-La Rocca HP, van Mechelen W, Puder JJ (2010). Effect of school based physical activity programme (KISS) on fitness and adiposity in primary schoolchildren: cluster randomised controlled trial. BMJ.

[4] Strong WB, Malina RM, Blimkie CJR, Daniels SR, Dishman RK, Gutin B, Hergenroeder AC, Must A, Nixon PA, Pivarnik JM (2005). Evidence based physical activity for school-age youth. J Pediatr.

[5] Troiano RP, Berrigan D, Dodd KW, Masse LC, Tilert T, McDowell M (2008). Physical activity in the United States measured by accelerometer. Med Sci Sports Exerc.

[6] Story M, Nanney MS, Schwartz MB (2009). Schools and obesity prevention: creating school environments and policies to promote healthy eating and physical activity. Milbank Q.

[7] Lounsbery MA, McKenzie TL, Trost S, Smith NJ (2011). Facilitators and barriers to adopting evidence-based physical education in elementary schools. J Phys Act Health.

[8] Cox L, Berends V, Sallis JE, St. John JM, McNeil B, Gonzalez M, Agron P (2011). Engaging school governance leaders to influence physical activity policies. J Phys Act Health.

[9] Eveland-Sayers BM, Farley RS, Fuller DK, Morgan DW, Caputo JL (2009). Physical fitness and academic achievement in elementary school children. J Phys Act Health.

[10] Kim H-YP, Frongillo EA, Han S-S, Oh S-Y, Kim W-K, Jang Y-A, Won H-S, Lee H-S, Kim S-H (2003). Academic performance of Korean children is associated with dietary behaviours and physical status. Asia Pac J Clin.

[11] Roberts CK (2010). Low aerobic fitness and obesity are associated with lower standardized test scores in children. J Pediatr.

[12] Sallis JF, McKenzie TL, Kolody B, Lewis M, Marshall S, Rosengard P (1999). Effects of health-related physical education on academic achievement: project SPARK. Res Q Exerc Sport.

[13] Trudeau F, Shephard RJ (2008). Physical education, school physical activity, school sports and academic performance. Int J Behav Nutr Phys Act.

[14] Institute of Medicine (2013). Educating the student body: taking physical activity and physical education to school.

[15] Singh A, Uijtdewilligen L, Twisk JW, van Mechelen W, Chinapaw MJ (2012). Physical activity and performance at school: a systematic review of the literature including a methodological quality assessment. Arch Pediatr Adolesc Med.

[16] McCullick BA, Baker T, Tomporowski PD, Templin TJ, Lux K, Isaac T (2012). An analysis of state physical education policies. J Teach Phys Educ.

[17] Carlson JA, Mignano AM, Norman GJ, McKenzie TL, Kerr J, Arredondo EM, Madanat H, Cain KL, Elder JP, Saelens BE (2014). Socioeconomic disparities in elementary school practices and children’s physical activity during school. Am J Health Promot.

[18] UCLA Center to Eliminate Health Disparities and Samuels & Associates (2007). Failing fitness: physical activity and physical education in schools. Funded by the California endowment.

[19] Kibbe DL, Hackett J, Hurley M, McFarland A, Schubert KG, Schultz A, Harris S (2011). Ten years of TAKE 10!®: integrating physical activity with academic concepts in elementary school classrooms. Prev Med.

[20] Mahar MT (2011). Impact of short bouts of physical activity on attention-to-task in elementary school children. Prev Med.

[21] Hatfield DP, Lynskey VM, Economos CD, Nichols ER, Whitman NB, Nelson ME (2016). Crowdsourcing innovative physical activity programs: active schools acceleration project case study. Transl J Am College Sports Med.

[22] Active Schools Acceleration Project [Internet]. Boston (MA): Active Schools Acceleration Project http://www.activeschoolsasap.org. Accessed 1 Aug 2016

[23] Franks AL, Kelder SH, Dino GA, Horn KA, Gortmaker SL, Wiecha JL, Simoes EJ (2007). School-based programs: lessons learned from CATCH, planet health, and not-on-tobacco. Prev Chronic Dis.

[24] Child and Adolescent Health Measurement Initiative 2005/06 NS-CSHCN. Health conditions and functional difficulties. Data Resource Center, supported by Cooperative Agreement 1‐U59‐MC06980‐01 from the U.S. Department of Health and Human Services, Health Resources and Services Administration (HRSA), Maternal and Child Health Bureau (MCHB). 2012. http://www.childhealthdata.org. Accessed 1 Aug 2016.

[25] Kuczmarski RJ, Ogden CL, Grummer-Strawn LM, Flegal KM, Guo SS, Wei R, Mei Z, Curtin LR, Roche AF, Johnson CL (2000). CDC growth charts: United States. Adv Data.

[26] Puyau MR, Adolph AL, Vohra FA, Butte NF (2002). Validation and calibration of physical activity monitors in children. Obes Res.

[27] National Oceanic and Atmospheric Association. National Climactic Data Center http://www.ncdc.noaa.gov/cdo-web/datasets. Accessed 1 Aug 2016.

[28] Evenson KR, Catellier DJ, Gill K, Ondrak KS, McMurray RG (2008). Calibration of two objective measures of physical activity for children. J Sports Sci.

[29] Taylor AF, Kuo FE (2009). Children with attention deficits concentrate better after walk in the park. J Atten Disord.

[30] van der Niet AG, Smith J, Oosterlaan J, Scherder EJA, Hartman E, Visscher C (2016). Effects of a cognitively demanding aerobic intervention during recess on children’s physical fitness and executive functioning. Pediatr Exerc Sci.

[31] Golden CJ, Freshwater SM (1978). Stroop color and word test.

[32] Buck SM, Hillman CH, Castelli DM (2008). The relation of aerobic fitness to stroop task performance in preadolescent children. Med Sci Sports Exerc.

[33] Hollar D, Messiah SE, Lopez-Mitnik G, Hollar TL, Almon M, Agatston AS (2010). Effect of a two-year obesity prevention intervention on percentile changes in body mass index and academic performance in low-income elementary school children. Am J Public Health.

[34] Chomitz VR, Slining MM, McGowan RJ, Mitchell SE, Dawson GF, Hacker KA (2009). Is there a relationship between physical fitness and academic achievement? Positive results from public school children in the northeastern United States. J Sch Health.

[35] Trost SG, McCoy TA, Vander Veur SS, Mallya G, Duffy ML, Foster GD (2013). Physical activity patterns of inner-city elementary schoolchildren. Med Sci Sports Exerc.

[36] Saunders RP, Pate RR, Felton G, Dowda M, Weinrich MC, Ward DS, Parsons MA, Baranowski T (1997). Development of questionnaires to measure psychosocial influences on children’s physical activity. Prev Med.

[37] Harter S. Self-perception profile for children. University of Denver. 2012. https://portfolio.du.edu/SusanHarter/page/44210. Accessed 1 Aug 2016.

[38] Harter S. The perceived competence scale for children. Child Dev. 1982;53(1):87–97.

[39] Cooper SB, Bandelow S, Nevill ME (2011). Breakfast consumption and cognitive function in adolescent schoolchildren. Physiol Behav.

[40] Cullen KW, Watson K, Zakeri I (2008). Relative reliability and validity of the Block Kids questionnaire among youth aged 10 to 17 years. J Am Diet Assoc.

[41] Rockett HR, Breitenbach M, Frazier AL, Witschi J, Wolf AM, Field AE, Colditz GA (1997). Validation of a youth/adolescent food frequency questionnaire. Prev Med.

[42] Smith C, Fila S (2006). Comparison of the Kid’s Block food frequency questionnaire to the 24‐hour recall in urban Native American youth. Am J Hum Biol.

[43] Block G, Murphy M, Roullet J, Wakimoto P, Crawford P, Block T (2000). Pilot validation of a FFQ for children 8–10 years. Fourth International Conference on Dietary Assessment Methods: 2000.

[44] USDA and USDHHS (2010). Dietary Guidelines for Americans, 2010.

[45] Todd V, Aaron LC, Jens CE, David BA (2009). Reliable prediction of insulin resistance by a school-based fitness test in middle-school children. Int J Pediatr Endocrinol.

[46] Leger L, Mercier D, Gadoury C, Lambert J (1988). The multistage 20 metre shuttle run test for aerobic fitness. J Sports Sci.

[47] Plowman SA, Meredith MD (2013). Fitnessgram/Activitygram reference guide.

[48] Lounsbery MAF, McKenzie TL, Morrow JR, Holt KA, Budnar RG (2013). School physical activity policy assessment. J Phys Act Health.

[49] Hubbard K, Economos CD, Bakun P, Boulos R, Chui K, Mueller MP, Smith K, Sacheck J (2016). Disparities in moderate-to-vigorous physical activity among girls and overweight and obese schoolchildren during school-and out-of-school time. Int J Behav Nutr Phys Act.

[50] Schwartz MB, Lund AE, Grow HM, McDonnell E, Probart C, Samuelson A, Lytle L (2009). A comprehensive coding system to measure the quality of school wellness policies. J Am Diet Assoc.

[51] Verstraete SJM, Cardon GM, De Clercq DLR, De Bourdeaudhuij IMM (2006). Increasing children’s physical activity levels during recess periods in elementary schools: the effects of providing game equipment. Eur J Public Health.

[52] Donnelly JE, Greene JL, Gibson CA, Sullivan DK, Hansen DM, Hillman CH, Poggio J, Mayo MS, Smith BK, Lambourne K (2013). Physical activity and academic achievement across the curriculum (A + PAAC): rationale and design of a 3-year, cluster-randomized trial. BMC Public Health.

[53] Hemming K, Girling AJ, Sitch AJ, Marsh J, Lilford RJ (2011). Sample size calculations for cluster randomised controlled trials with a fixed number of clusters. BMC Med Res Methodol.

[54] Singh GK, Siahpush M, Kogan MD (2010). Rising Social Inequalities in US childhood obesity, 2003–2007. Ann Epidemiol.

[55] Fairchild AJ, MacKinnon DP (2009). A general model for testing mediation and moderation effects. Prev Sci.

[56] Belcher BR, Berrigan D, Dodd KW, Emken BA, Chou CP, Spruijt-Metz D (2010). Physical activity in US youth: effect of race/ethnicity, Age, gender, and weight status. Med Sci Sports Exerc.

[57] Siceloff ER, Wilson DK, Van Horn L (2014). A longitudinal study of the effects of instrumental and emotional social support on physical activity in underserved adolescents in the ACT trial. Ann Behav Med.

[58] Wilson DK, Lawman HG, Segal M, Chappell S (2011). Neighborhood and parental supports for physical activity in minority adolescents. Am J Prev Med.

